# Tumors carrying *BRAF*-mutations over-express NAMPT that is genetically amplified and possesses oncogenic properties

**DOI:** 10.1186/s12967-022-03315-9

**Published:** 2022-03-10

**Authors:** Valentina Audrito, Enrico Moiso, Filippo Ugolini, Vincenzo Gianluca Messana, Lorenzo Brandimarte, Ilaria Manfredonia, Simonetta Bianchi, Francesco De Logu, Romina Nassini, Anna Szumera-Ciećkiewicz, Daniela Taverna, Daniela Massi, Silvia Deaglio

**Affiliations:** 1 Laboratory of Functional Genomics, Department of Medical Sciences, University of Turin, Via Nizza, 52, 10126 Torino, Italy; 2 Koch Institute for Integrative Cancer Research, Massachusetts Institute of Technology, Cambridge, MA USA; 3 Broad Institute of Harvard and MIT, Cambridge, MA USA; 4 Department of Health Sciences, University of Florence, Florence, Italy; 5 Department of Pathology and Laboratory Medicine, Maria Sklodowska-Curie National Research Institute of Oncology, Warsaw, Poland; 6 Diagnostic Hematology Department, Institute of Hematology and Transfusion Medicine, Warsaw, Poland; 7 Present Address: Department of Molecular Biotechnology and Health Sciences, University of Torino, Torino, Italy

**Keywords:** NAMPT, Oncogene, Transformation, BRAF oncogenic signaling, MAPK, NAMPT inhibitors

## Abstract

**Background:**

Nicotinamide phosphoribosyltransferase (NAMPT), the rate-limiting enzyme in nicotinamide adenine dinucleotide (NAD) biosynthesis, is up-regulated in several cancers, including metastatic melanoma (MM). The *BRAF* oncogene is mutated in different cancer types, among which MM and thyroid carcinoma (THCA) are prominent. Drugs targeting mutant BRAF are effective, especially in MM patients, even though resistance rapidly develops. Previous data have linked NAMPT over-expression to the acquisition of BRAF resistance, paving the way for therapeutic strategies targeting the two pathways.

**Methods:**

Exploiting the TCGA database and a collection of MM and THCA tissue microarrays we studied the association between *BRAF* mutations and NAMPT expression. *BRAF* wild-type (wt) cell lines were genetically engineered to over-express the *BRAF* V600E construct to demonstrate a direct relationship between over-activation of the BRAF pathway and NAMPT expression. Responses of different cell line models to NAMPT (i)nhibitors were studied using dose–response proliferation assays. Analysis of *NAMPT* copy number variation was performed in the TCGA dataset. Lastly, growth and colony forming assays were used to study the tumorigenic functions of NAMPT itself.

**Results:**

The first finding of this work is that tumor samples carrying *BRAF*-mutations over-express NAMPT, as demonstrated by analyzing the TCGA dataset, and MM and THC tissue microarrays. Importantly, *BRAF* wt MM and THCA cell lines modified to over-express the *BRAF* V600E construct up-regulated NAMPT, confirming a transcriptional regulation of NAMPT following BRAF oncogenic signaling activation. Treatment of *BRAF*-mutated cell lines with two different NAMPTi was followed by significant reduction of tumor growth, indicating NAMPT addiction in these cells. Lastly, we found that several tumors over-expressing the enzyme, display *NAMPT* gene amplification. Over-expression of NAMPT in BRAF wt MM cell line and in fibroblasts resulted in increased growth capacity, arguing in favor of oncogenic properties of NAMPT.

**Conclusions:**

Overall, the association between *BRAF* mutations and NAMPT expression identifies a subset of tumors more sensitive to NAMPT inhibition opening the way for novel combination therapies including NAMPTi with BRAFi/MEKi, to postpone and/or overcome drug resistance. Lastly, the over-expression of NAMPT in several tumors could be a key and broad event in tumorigenesis, substantiated by the finding of *NAMPT* gene amplification.

**Supplementary Information:**

The online version contains supplementary material available at 10.1186/s12967-022-03315-9.

## Background

Nicotinamide phosphoribosyltransferase (NAMPT), the rate-limiting enzyme in nicotinamide adenine dinucleotide (NAD) biosynthesis, is over-expressed in a broad range of tumors [1–4]. Acting both at the intracellular and extracellular levels, NAMPT exerts a direct role on tumor cells increasing tumor aggressiveness and modifying interactions with the tumor microenvironment [2, 3, 5]. In general, NAMPT performs as a negative prognostic marker, with over-expression correlating with shorter time to treatment and inferior overall survival [2, 3]. Over-expression can be determined at mRNA and protein levels detected in patients by quantitative PCR and immunohistochemistry. In addition, NAMPT may also be quantified as an extracellular protein released in the circulation and detected using ELISA assay. Even though inhibiting NAMPT was proposed as a therapeutic strategy for cancer patients, NAMPT (i)nhibitors showed no objective tumor remission in early clinical trials. Beside the pharmacological limitations of old drugs and their potential toxicity, one of the main aspects leading to the failure of NAMPTi was attributed to the concomitant expression of other NAD-biosynthetic enzymes (NBEs) that could compensate for NAMPT inhibition, overcoming it [6, 7]. It is therefore becoming increasingly clear that it is necessary to carefully select tumors that are uniquely addicted to NAMPT activity to generate NAD and to potentially combine these inhibitors with other targeted therapies.

Using metastatic melanoma (MM) as disease model, we and others previously demonstrated that NAMPT is over-expressed during BRAFi resistance and patient relapse/progression, becoming a driver of targeted-therapy resistance [8–11]. Moreover, BRAFi-resistant MM cells are uniquely sensitive to NAMPTi in vitro*,* and in vivo [9], supporting a molecular connection between NAMPT and BRAF oncogenic pathways. The *NAMPT* promoter contains several binding sites for known transcription factors (TFs), among which cAMP response element-binding protein (CREB), signal transducer and activator of transcription (STATs), hypoxia inducible factor (HIF), nuclear factor-kB (NF-κB), ERK and MYC, all downstream of the RAS/BRAF pathway. However, the relationship between NAMPT over-expression in cancer and the activation of oncogenic pathways remains incompletely understood.

Activating mutations in *BRAF* have been described in MM (˜50%), thyroid (THCA, 30–50%), colorectal (CRC, 10%), non-small cell lung (NSCLC, 3%) cancers and in hairy cell leukemia (HCL, 100%) [12]. Initially described in 2002, *BRAF* mutations lead to constitutive activation of the kinase BRAF and, consequently, of the RAF-MEK-ERK signaling cascade, promoting cell proliferation and survival, while inhibiting apoptosis, ultimately driving cancer growth [13, 14]. As a result, several selective BRAFi are now being clinically used, also in combination with MEKi, with impressive results, particularly for patients with MM [15–17]. However, the clinical benefit of this treatment is inevitably limited by the onset of resistance [18, 19], underlining the need for additional therapeutic targets and combination therapies.

The aim of this work is to understand the connection between NAMPT expression and *BRAF* mutations and investigate the potential oncogenic activity of NAMPT itself. Here we demonstrate that mutations in the *BRAF* oncogene, as well as in other genes belonging to MAPK pathway, positively correlate with NAMPT expression. *BRAF*-mutated tumors become strictly dependent on this enzyme for NAD generation and are therefore more sensitive to NAMPTi. Based on these pre-clinical results, it may be possible to envisage using a combination of NAMPTi and BRAFi/MEKi in the treatment of patients with this type of tumors. In addition, we revealed oncogenic properties of NAMPT, independently of *BRAF* mutations, substantiated by the finding of gene amplification in human tumors and promotion of cell growth capacity upon NAMPT over-expression.

## Materials and methods

### Reagents

FK866 was from Selleckchem (Munich, DE), and GMX1778 was from Sigma (Milan, IT).

### TCGA analysis

Genomic data shown in this paper are in whole or part based upon data generated by The Cancer Genome Atlas (TCGA) Research Network (http://cancergenome.nih.gov/). Details are included in Additional file 1: Methods.

### Immunohistochemistry staining

#### Tissues samples

Formalin-fixed and paraffin-embedded (FFPE) MM representative samples (n = 165) were collected from patients diagnosed and treated in Maria Sklodowska-Curie National Research Institute of Oncology, Warsaw, Poland. All cases were molecularly characterized, as previously described [20]. FFPE THCA (thyroid papillary carcinoma) representative samples (n = 84) were collected from the section of Pathological Anatomy, Department of Health Sciences of the University of Florence. More detailed are included in Additional file 1: Methods.

#### High-density tissue microarrays (TMAs)

High-density TMAs were constructed from archival MM FFPE, including 3 representative 1.0 mm cores from each case using the automatic system (TMA Grand Master, 3DHistech*). In all TMAs positive and negative controls were enclosed (tonsil, testis, liver, appendix, and normal skin).

#### Immunohistochemistry

All MM samples and TMA were stained with the NAMPT antibody (mouse monoclonal, clone OMNI379, 20A-0034, 1:300, Adipogen, San Diego, CA), as described in Additional file 1: Methods. Tissues were digitally scanned at a 400× magnification (Aperio AT2 platform, Leica Biosystems, Wetzlar, DE) for the evaluation using a semi-quantitative NAMPT H-score analysis as described in Additional file 1: Methods.

### Cell culture

*BRAF* V600E-mutated cell lines were M14 and A375 (MM) and 8305C and BHT-101 (THCA), while *BRAF* WT lines were MEL505 and Mewo (MM) and CAL-62 and ML-1 (THCA), all purchased from DSMZ (Braunschweig, DE) or ATCC (LGC Standards S.r.l. Italy distributor Sesto San Giovanni, Milan, IT), as for NIH-3T3. Cells were cultured as described in Additional file 1: Methods.

### Transduction of *BRAF *WT cell lines with a *BRAF* V600E construct

The Tween-Myc-*BRAF* V600E-GFPtag lentivirus vector [21], kindly gift by Prof. Tiacci and Dr. Pettirossi (University and Hospital of Perugia, Perugia, IT), was used to induce over-expression of *BRAF* V600E in cells that naturally did not harbor *BRAF* V600E mutation. An empty Tween-GFPtag vector was used as control. GFP^+^ cells were flow sorted (FACSAriaIII, BD Biosciences) and expanded.

### NAMPT over-expression

The previously obtained lentivirus vector for NAMPT-GFPtag [9] was used to over-express NAMPT in Mewo cell line (*BRAF* wt) and in NIH-3T3. An empty GFPtag vector was used as control. GFP^+^ cells were flow sorted using FACSAriaIII and expanded for further experiments.

### RNA extraction and quantitative real‐time PCR (qRT‐PCR)

RNA extraction and qRT-PCR were performed as described in [8], using commercially available primers (TaqMan Gene Expression Assays; Thermo Fisher Scientific, Rodano MI, IT) listed in Additional file 1: Methods.

### Western blot analysis

Western blot analysis to evaluate proteins expression was performed as described previously [9]. Antibodies used were listed in Additional file 1: Methods.

### Confocal microscopy

Cells were cultured on glass cover slips in 24-well plates for 24 h. Staining with anti-NAMPT antibody (A300-779A, 1:200, Bethyl Laboratories, Montgomery, TX) was performed as described [9]. Slides were analyzed using a TCS SP5 laser scanning confocal microscope with an oil immersion 63× objective and acquired with LAS AF software (both from Leica Microsystems, Milan, IT). Images were processed with Photoshop (Adobe Systems, San Jose, CA). Pixel intensity was calculated using the ImageJ software (http://rsbweb.nih.gov/ij/).

### Colony formation assay

Cells (500/well) were seeded into 6-well plates and cultured for 10–12 days in a complete medium. Cells were then fixed with 4% PFA [10 min, room temperature (RT)] and stained with crystal violet (20 min, RT, in the dark). The percentage of occupied colony areas was calculated with ImageJ/Fiji software.

### Cell viability

Cell viability was evaluated after 72 h of treatment using an MTT assay kit (Thermo Fisher Scientific). Each sample was measured in triplicate and repeated at least three times.

### Statistical analysis

Statistical comparisons were performed using Graph Pad Prism version 7.0 (Graph Pad Software Inc., La Jolla, CA) Statistical significance was determined by two-tailed Mann–Whitney U or unpaired/paired Student’s t test and one-way ANOVA test. Unless otherwise indicated, data in the Figures are presented as the mean ± SEM.

Statistical TCGA analyses were performed using R v3.5.1 (http://www.r-project.org) and Rstudio v1.1.463 (http://www.rstudio.com/). Correlation between gene mutation status and *NAMPT* expression has been calculated by both Kolmogorov–Smirnoff test for difference between the cumulative distributions and Mann–Whitney test for difference in the expression rank.

Correlations between NAMPT expression levels and other genes or *NAMPT* Copy Number Alteration (CNA) segment gain or loss (SGOL) scores have been calculated by means of Pearson correlation coefficient. Difference between mean NAMPT SGOL from 0 has been calculated by means of one sample t-test. The statistical analysis of the cumulative distribution function of CNA profile of primary and metastatic derived melanoma samples has been performed by means of a Kolmogorov–Smirnov test.

#### Array-CGH (aCGH) and SGOL scores

CNA SGOL scores at the single gene level were generated by means of cghMCR [22], DNAcopy [23] and CNTools [24] Bioconductor packages, starting from CGH array segmented data. CghMCR package allows the calculation of SGOL starting by segmented data, by means of a modified version of GISTIC algorithm. The segment function of DNAcopy package is used to segment the normalized data so that chromosome regions with the same copy number have the same segment mean values. Then, by means of CNTools, getRS function, the data returned by segment are organized in a matrix format. SGOL function of cghMCR is ultimately used to compute the SGOL scores for genes.

For all statistical tests, the 0.05 level of confidence was accepted for statistical significance. Significance was represented as: *p ≤ 0.05, **p ≤ 0.01, ***p ≤ 0.001, ****p ≤ 0.0001.

## Results

### NAMPT expression levels are directly correlated to *BRAF* oncogenic mutations in patients

**Fig. 1:**
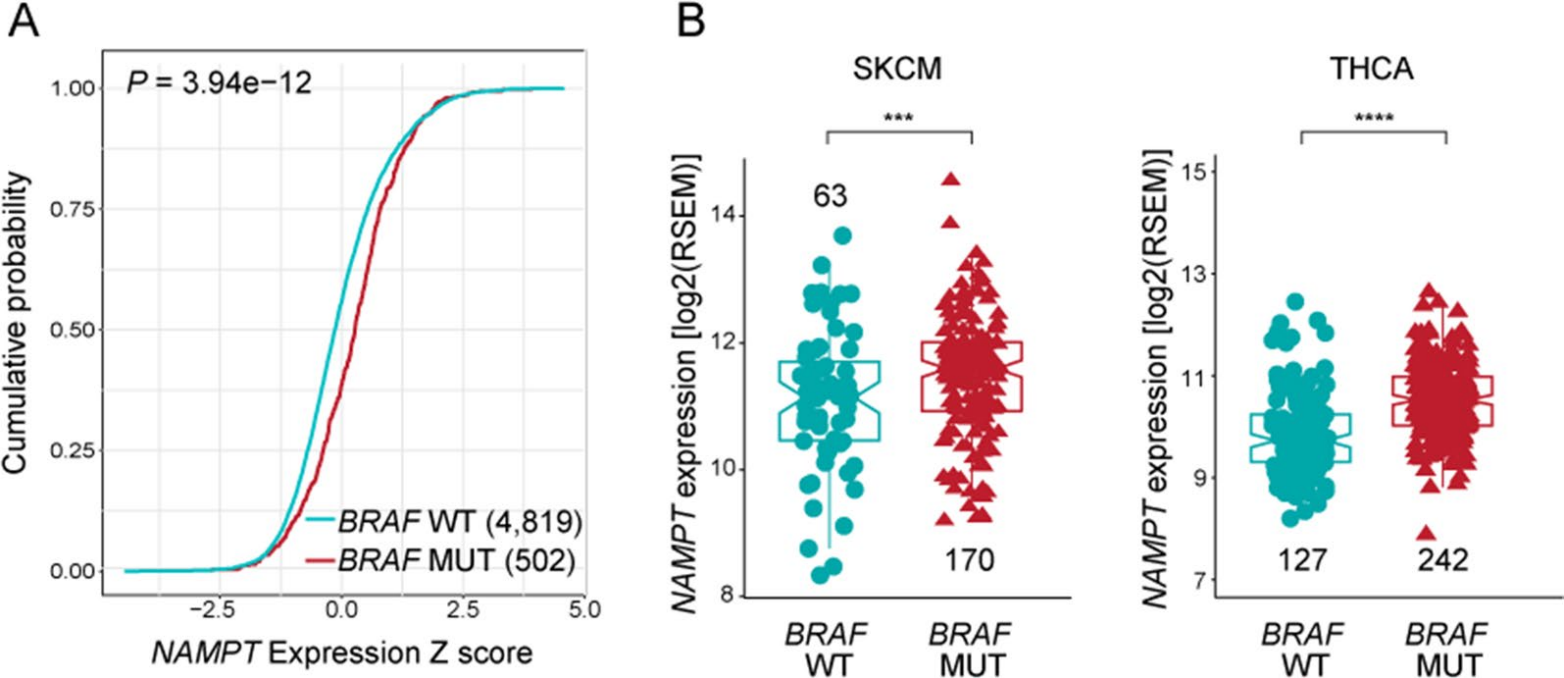
*NAMPT* expression is associated with *BRAF* mutations in TCGA cohort. **A** Cumulative distribution function (cdf) plot of *NAMPT* expression in *BRAF* wild-type (WT, in cyan) and *BRAF* mutated (MUT, in red) tumor samples from the TCGA cohort. The shift toward the right for the cdf indicates *NAMPT* increased expression in *BRAF* mutated samples. N of samples for each group are indicated in the brackets. Statistical significance was calculated using Kolmogorov–Smirnoff test. **B** Boxplots representing *NAMPT* expression levels in *BRAF* WT vs.* BRAF* MUT SKCM and THCA samples of the TCGA cohort. N of samples in each group are indicated (excluding from the analysis *NRAS* and *C-KIT* mutated samples). Statistical significance was calculated using Mann–Whitney test. Significance was represented as: *p ≤ 0.05, **p ≤ 0.01, ***p ≤ 0.001, ****p ≤ 0.0001

To highlight a possible association between NAMPT expression and *BRAF* mutations in human cancers, we analyzed the TCGA database showing that *BRAF*-mutated tumors (MUT, in red) display significantly higher levels of *NAMPT*, as reported by cumulative distribution function (*P* = 3.94 × 10^–12^, Fig. 1A). This observation was obtained globally considering all cancers, notwithstanding intrinsic tumor heterogeneity (Additional file 1: Fig. S1A). We then focused our analysis on melanoma [Skin Cutaneous Melanoma (SKCM) TCGA cohort] and THCA, as tumors with significant percentages of *BRAF* mutations (Additional file 1: Fig. S1B) [25, 26]. All annotated *BRAF* mutations were considered, with V600E being the most frequently represented (131/186, 70% for SKCM and 242/254, 95% in THCA). In the case of SKCM 20 cases (11%) were characterize by the V600K, a second activating mutation, while the remaining 35 cases (19%) presented with other BRAF mutations (Additional file 1: Fig. S1C). In some instances (12/254, 5% for THCA and 16/186, 9% for SKCM), the tumors displayed additional mutations in the RAS pathway (*NRAS*, *NF1,* and *CKIT*) and were discarded from the analyses presented in Fig. 1A. In both these tumor types, *NAMPT* expression was significantly higher in patients harboring *BRAF* mutations [SKCM: 170 *BRAF*-MUT vs. 63 *BRAF*-WT, *P* = 0.0006, and THCA: 242 *BRAF*-MUT vs. 127 *BRAF*-WT, *P* = 10^–15^ (Fig. 1B)].

### *BRAF*-mutated tumors over-express NAMPT at protein levels

The positive correlation between NAMPT expression and *BRAF* mutations in patients was then confirmed at protein level by comparing NAMPT staining in *BRAF*-WT and *BRAF*-mutated samples in MM tissue microarrays (TMA) and THCA whole slide sections.

**Fig. 2:**
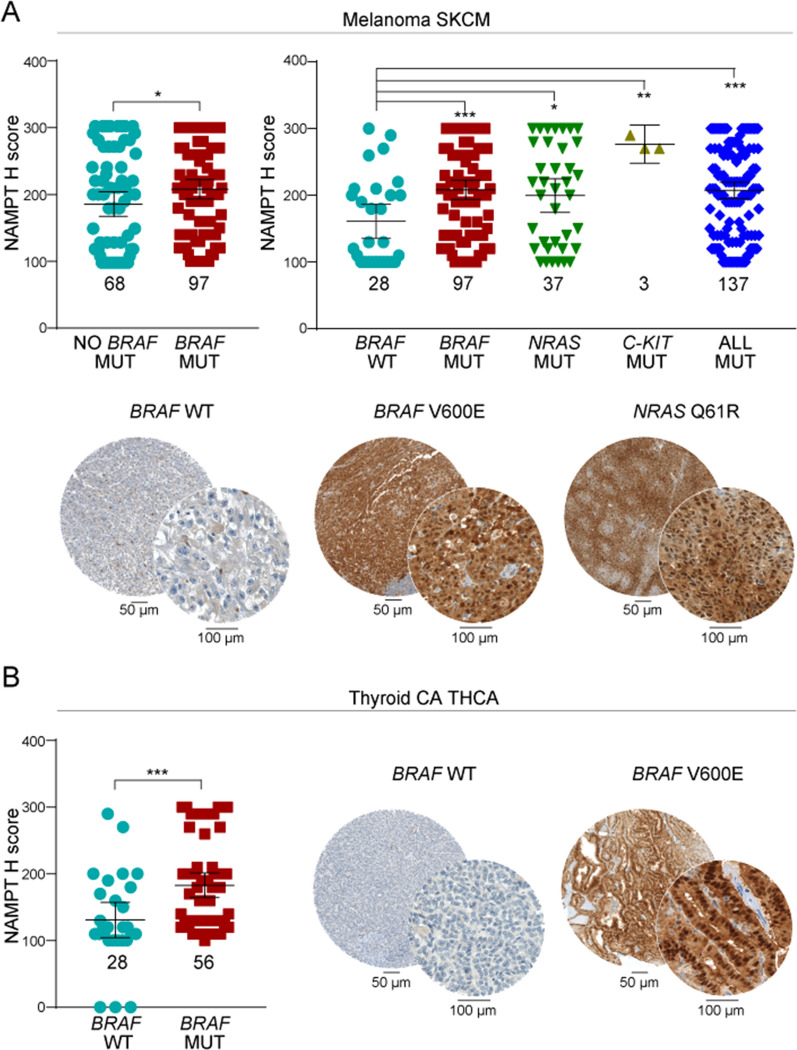
NAMPT protein levels are increased in *BRAF*-mutated vs. BRAF WT melanoma and thyroid carcinomas. **A** Dot plots representing NAMPT protein levels [H-score, defined by immunohistochemistry (IHC) staining] on tissue microarrays (TMAs) in metastatic melanoma (MM) grouped in *BRAF* WT, *BRAF* mutated (MUT), *NRAS* MUT and *C-KIT* MUT. N of samples in each group are indicated. Statistical significance was calculated using Mann–Whitney test. The line in the dot plot defines the median and the error bars define the interquartile range. Below the graphs, representative immunohistochemistry (IHC) stainings for NAMPT in *BRAF* WT, *BRAF* V600E, and *NRAS* Q61R MM cases. Scale bars are reported under the images. **B** Dot plot representing H-score of NAMPT protein levels on THCA whole slide sections grouped in *BRAF* WT and *BRAF* V600E. N of samples in each group are indicated. Statistical significance was calculated using Mann–Whitney test. The line in the dot plot defines the median and the error bars define the interquartile range. On the right, representative IHC staining for NAMPT in THCA cases. Scale bars are reported under images. Significance was represented as: *p ≤ 0.05, **p ≤ 0.01, ***p ≤ 0.001, ****p ≤ 0.0001

In a MM cohort including 68 *BRAF*-WT and 97 *BRAF*-MUT tissue samples, a statistically significant increase of NAMPT staining, expressed as H score, was highlighted in *BRAF*-mutated cases (*P* < 0.05, Fig. 2A, left graph). Notably, when considering only the true WT cases (n = 28, i.e., excluding those with *NRAS* and *c-KIT* mutations), statistical significance markedly increased in all comparisons [28 WT cases vs. 97 *BRAF*-MUT (n = 97, *P* = 0.0008), or vs.* NRAS*-MUT (n = 37, *P* = 0.01), or vs.* c-KIT*-MUT (n = 3, *P* = 0.005), or vs. a combination of all mutations in *BRAF*, *NRAS* and *c-KIT* (n = 137, *P* = 0.0007)] (Fig. 2A, graph on the right). IHC analysis of NAMPT expression highlighted a strong reactivity for NAMPT in cases harboring these mutations (Fig. 2A, IHC representative images).

In THCA, only *BRAF* V600E mutation was analyzed, as it is the most commonly observed genetic abnormality in this tumor [27]. Consistent with MM samples, NAMPT staining was significantly increased in *BRAF*-MUT (n = 56) compared to *BRAF*-WT (n = 28) THCA cases (*P* = 0.0007, Fig. 2B).

Overall, these results demonstrate a positive and statistically significant correlation between *BRAF* mutational status and NAMPT mRNA and protein expression levels in patients’ tumors. In addition, mutations in other genes that insist on the MAPK/ERK pathway, such as *NRAS* and *c-KIT*, also lead to NAMPT over-expression.

### Activation of the BRAF oncogenic pathway leads to up-regulation of NAMPT expression in MM and THCA cell lines

**Fig. 3:**
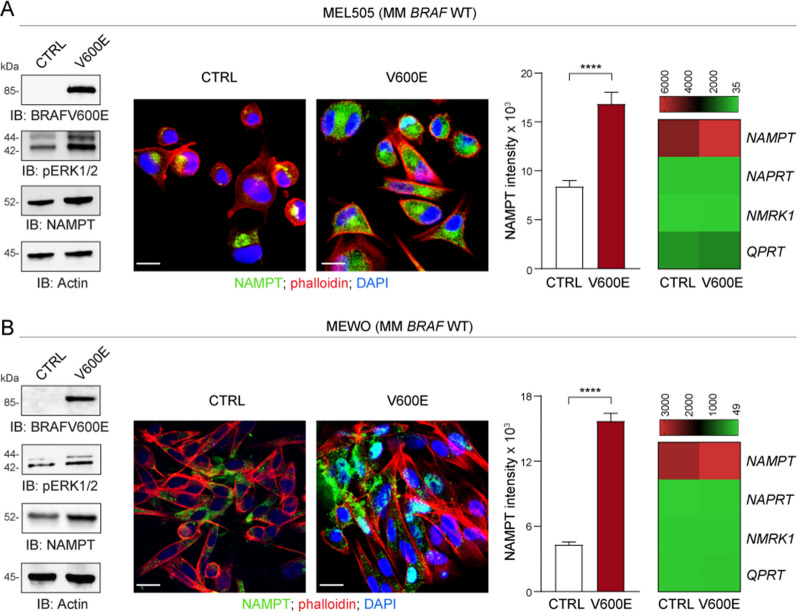
BRAF oncogenic pathway activation selectively up-regulates NAMPT expression in MM. **A**, **B** *BRAF* WT MM cell lines MEL-505 (**A**) and Mewo (**B**) were infected with a lentivirus carrying the *BRAF* V600E construct. A control (CTRL) vector was used as comparison. Panels on the left show representative western blot analysis of BRAF V600E, phosphorylated (p)ERK1/2 and NAMPT expression. Actin was used as loading control. In the middle of the Figure NAMPT expression (green fluorescence), evaluated by confocal staining (original magnification ×63, scale bar: 25 µm). Histogram plots represent cumulative data of NAMPT expression (intensity) measured from at least 3 different fields of 3 pictures of 3 independent confocal experiments comparing CTRL (white bar) vs. V600E cells (red bar). Statistical significance was calculated using Mann–Whitney test. Heatmaps on the right show cumulative data of qRT-PCR analysis of the expression of NBEs i.e., *NAMPT*/*NAPRT*/*NMRK1*/*QPRT* (mean of at least 5 independent experiments) in all cell line variants. Data in the Figure are presented as the mean ± SEM. Significance was represented as: *p ≤ 0.05, **p ≤ 0.01, ***p ≤ 0.001, ****p ≤ 0.0001

**Fig. 4:**
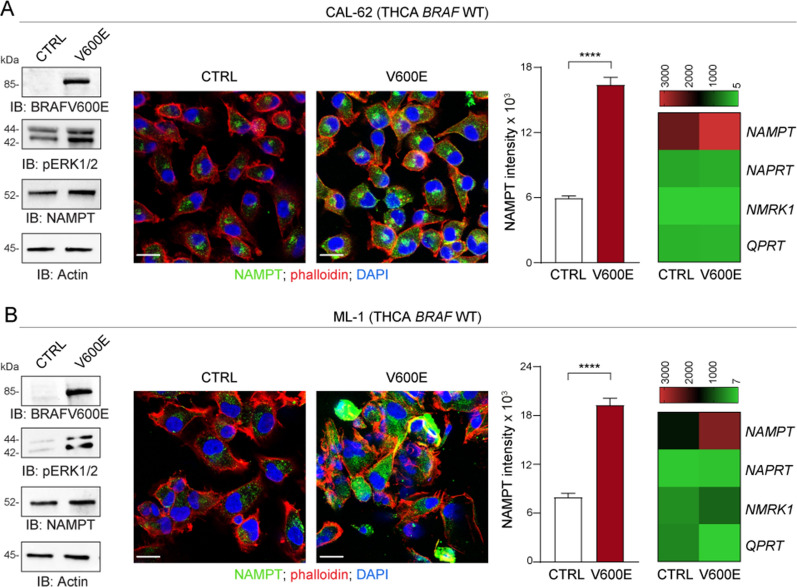
BRAF oncogenic pathway activation selectively up-regulates NAMPT expression in THCA. **A**, **B** *BRAF* WT THCA cell lines CAL-62 (**A**) and ML-1 (**B**) were infected with a lentivirus carrying the *BRAF* V600E construct. A control (CTRL) vector was used as comparison. Panels on the left show representative western blot analysis of BRAF V600E, phosphorylated (p)ERK1/2 and NAMPT expression. Actin was used as loading control. In the middle of the Figure NAMPT expression (green fluorescence), evaluated by confocal staining (original magnification ×63, scale bar: 25 µm). Histogram plots represent cumulative data of NAMPT expression (intensity) measured from at least 3 different fields of 3 pictures of 3 independent confocal experiments comparing CTRL (white bar) vs. V600E cells (red bar). Statistical significance was calculated using Mann–Whitney test. Heatmaps on the right show cumulative data of qRT-PCR analysis of the expression of NBEs i.e., *NAMPT*/*NAPRT*/*NMRK1*/*QPRT* (mean of at least 5 independent experiments) in all cell line variants. Data in the Figure are presented as the mean ± SEM. Significance was represented as: *p ≤ 0.05, **p ≤ 0.01, ***p ≤ 0.001, ****p ≤ 0.0001

To have a formal validation of the direct relationship between over-activation of the BRAF oncogenic pathway and NAMPT expression, two *BRAF*-WT MM cell lines (MEL505 and Mewo, Fig. 3A, B) and two *BRAF*-WT THCA cell lines (CAL-62 and ML-1, Fig. 4A, B) were infected with a lentivirus carrying the *BRAF* V600E construct, leading to the expected constitutive activation of the pathway, with increased baseline ERK phosphorylation (p-ERK) levels (Figs. 3 and 4). As demonstrated by RNA and protein analysis, NAMPT expression in these cells was significantly increased compared to control cells infected with an empty vector (Figs. 3 and 4). Up-modulation was specific for NAMPT, while the other three NBEs, i.e., *NAPRT*, *NMRK1,* and *QPRT*, were unaffected by infection with *BRAF* V600E (Figs. 3 and 4, heatmaps on the right). These findings indicate that the BRAF oncogenic pathway is directly responsible for NAMPT transcriptional and protein over-expression.

### *BRAF*-mutated tumors are more sensitive to NAMPTi compared to the *BRAF*-WT ones

**Fig. 5:**
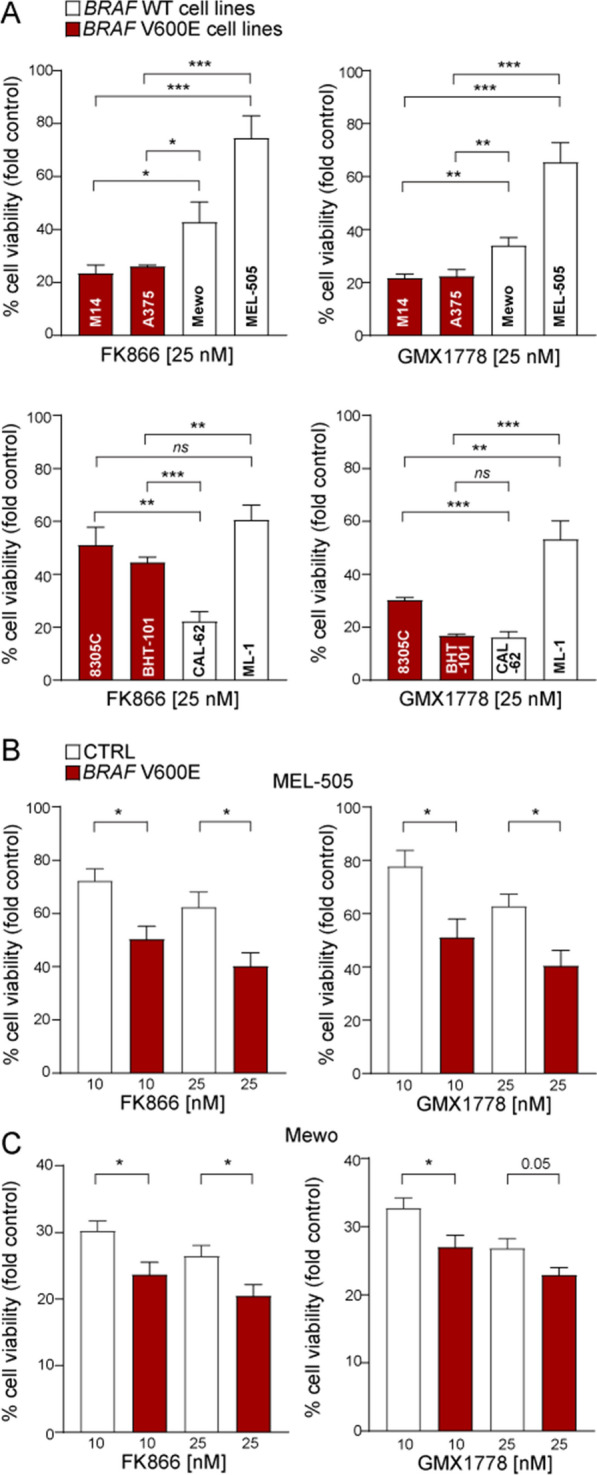
Response to NAMPT inhibition in MM and THCA cell lines according to *BRAF* mutations. **A** Short-term proliferation assay comparing sensitivity to NAMPTi (FK866 and GMX1778 at 25 nM, 72 h of treatment) of MM *BRAF*-mutated vs. WT cell lines (upper graphs) and THCA *BRAF*-mutated vs. WT cell lines (lower graphs). Data represent the average and SEM of at least three biological replicates. Unpaired t test was used to calculate the statistical difference of sensitivity between *BRAF* WT vs. mutated cell lines. **B**, **C** Proliferation assay comparing sensitivity to NAMPTi in MEL-505 (**B**) and Mewo (**C**) infected with a CTRL or *BRAF* V600E construct. Data represent the average and SEM of at least three biological replicates. Significance was represented as: *p ≤ 0.05, **p ≤ 0.01, ***p ≤ 0.001, ****p ≤ 0.0001

We then asked whether the direct impact of the BRAF oncogenic pathway on NAMPT expression could reflect a different sensitivity to NAMPTi between WT and *BRAF*-mutated cell lines. To understand if *BRAF*-mutated tumors rely on NAMPT activity and could therefore respond to NAMPTi, we compared the effects of two known NAMPTi (i.e., FK866 and GMX1778) in the *BRAF*-mutant *vs BRAF*-WT cell lines. Dose–response proliferation assays in *BRAF*-mutated MM cell lines (M14 in orange, A375 in red) and in *BRAF*-mutated THCA (BHT-101 in blue, 8305C in light blue), as shown in Additional file 1: Fig. S2A, demonstrated a significant response in term of reduction of cell viability in response to both NAMPTi (at doses < 10 nM) after 72 h in all *BRAF*-mutated cell lines compared to control cells. Selecting an intermediate dose of both NAMPTi (i.e., 25 nM), also previously employed in MM model [9], we obtained more sensitivity to NAMPTi in MM cell lines (M14, A375) carrying *BRAF* mutations than in the WT ones (MEL-505, Mewo, Fig. 5A, upper graphs). The same results were obtained in THCA cell lines (8305C, BHT-101 vs. ML-1). Unexpectedly CAL-62 cell line showed an high sensitivity to both NAMPTi (Fig. 5A, lower graphs), even if it is *BRAF*-WT. Analyzing differences in the mutational status of this THCA cell line compared to the other we noticed that CAL-62 cells carry an activating mutation in the *KRAS* (G12R, c.34G>C) oncogene, the third most common *KRAS* mutation in pancreatic ductal adenocarcinoma (PDAC) [28]. This kind of mutation belongs to the genetic alterations in epidermal growth factor receptor (EGFR) pathway, like *BRAF* and *NRAS,* suggesting that also different mutations in upstream genes that insist on the same oncogenic signaling pathway may influence responses to NAMPTi, making these cells highly sensitive to NAMPT targeting.

Consistently, using *BRAF*-WT MM cells infected with a lentivirus carrying the *BRAF* V600E construct, sensitivity to NAMPTi is markedly increased (red vs. white bars, Fig. 5B, C).

Overall, these data clearly demonstrate that *BRAF*-mutated tumors are highly sensitive to NAMPTi, highlighting a strong rationale to combine them with BRAFi/MEKi.

### *NAMPT* is genetically amplified in cancers, including in patients with melanoma

NAMPT over-expression is a feature of several cancers, not only the *BRAF*-mutated ones. Moreover, the observation that NAMPT-regulated NAD metabolism impact many tumors’ cellular pathways is well established by several studies in these years [3, 5]. However, the role of NAMPT in tumorigenesis is not fully understood, prompting the question of whether NAMPT itself has oncogenic properties.

**Fig. 6:**
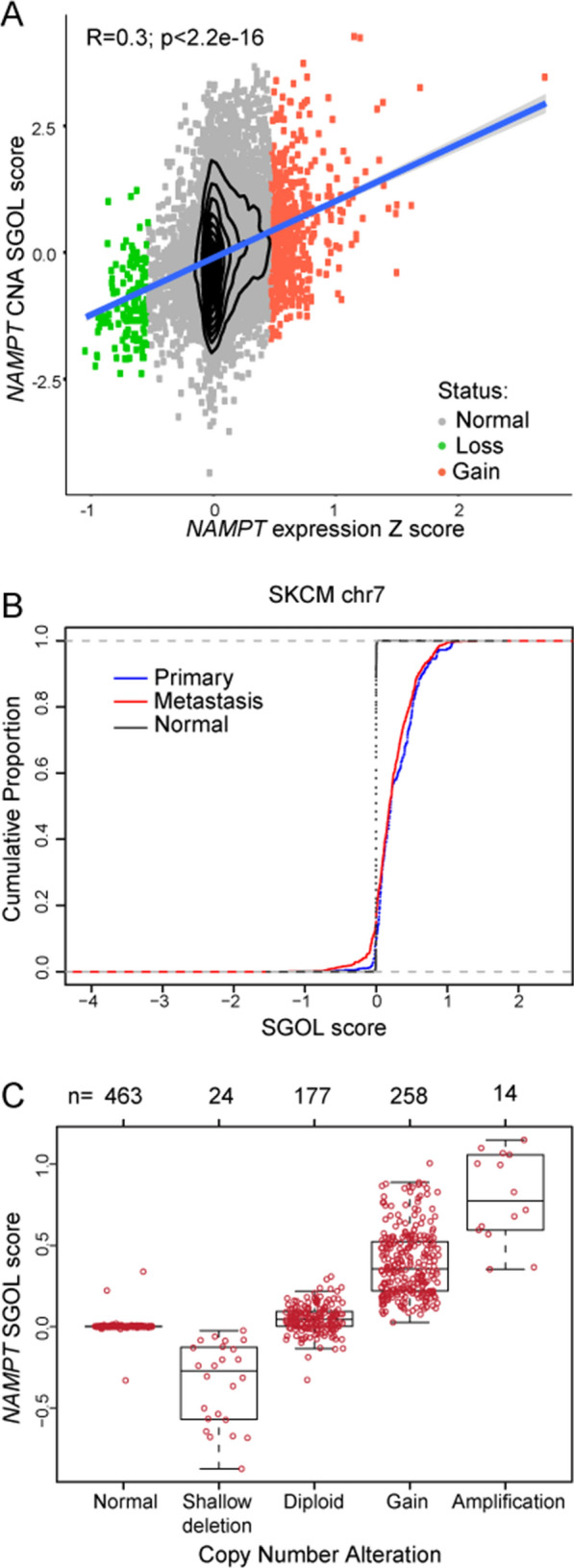
*NAMPT* is genetically amplified in cancers, specifically in melanoma. **A** Scatter plot showing a positive correlation between *NAMPT* expression levels (Z score) and copy number alteration (CNA) SGOL scores across all TCGA samples for which paired CNA and expression values were present. R: Pearson coefficient, p: p-value. **B** Cumulative distribution function (cdf) plot of SGOL score for all coding genes on chr7 in primary (blue), metastatic (red) and matched blood-derived normal (black) samples from TCGA SKCM cohort. We can notice the shift toward left for the cdf of primary and metastatic derived samples respect to normal samples indicating amplification of the *NAMPT* gene. **C** Boxplot representing the quantification (n) and categorization of *NAMPT* SGOL scores from SKCM and control-matched samples

Gain-of-function mutations or genetic amplification convert proto-oncogenes into oncogenes. Therefore, we looked for *NAMPT* mutations and copy number alterations (CNA) interrogating the TCGA datasets. Very few mutations in *NAMPT* were found in all tumors, less than 1%, confirming previous results [29]. On the contrary, we revealed a significant *NAMPT* CNA at the 7q22 locus where *NAMPT* mapped in all tumor samples, assessed by array CGH and measured as Segments of Gain Or Loss (SGOL) score (Fig. 6A, P < 2.2e−16). *NAMPT* CNA positively correlates with its expression, across 33 different tumor types. Notably, the average *NAMPT* SGOL score across all tumor types in the TCGA cohort is always positive (Additional file 1: Fig. S3A), suggesting a general tendency toward amplification. *NAMPT* expression levels (Additional file 1: Fig. S3A, lower panels) parallel its CNA levels (Additional file 1: Fig. S3A, higher panel).

Focusing again on melanoma, we observed amplification of the whole chromosome 7 in melanoma samples, at variance with normal subjects (black line) showing no CNA in that chromosome, in line with previous results [30]. Interestingly, lower SGOL scores were more frequently observed in metastatic samples (red line), and higher scores were more frequently found in primary melanoma samples (blue line, P < 2.2e−16), as depicted in Fig. 6B, suggesting that amplification of chromosome 7 is an early event in melanoma development. Analyzing only *NAMPT* gene, it is evident that it is more frequently subjected to gain/amplification in melanoma compared to control-matched samples (Fig. 6C). No statistical differences were observed in the *NAMPT* SGOL score between primary and metastatic samples (data not shown). These observations are consistent with the possibility of a gain-of-function role for NAMPT in tumors, and specifically in melanoma.

### NAMPT over-expression in *BRAF*-WT melanoma cell line and in fibroblasts NIH-3T3 increases cell growth and the colony-formation capacity

**Fig. 7:**
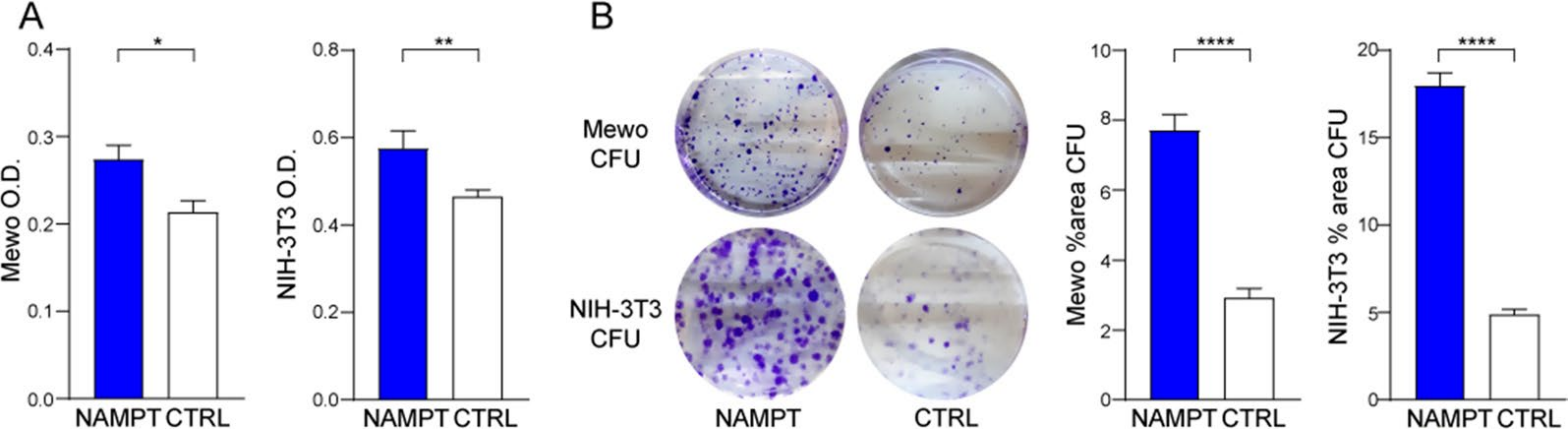
NAMPT possesses oncogenic properties. **A** Basal cell growth of NAMPT over-expressing (NAMPT) vs. control GFP (CTRL) cells, as measured by the MTT assay. Data are from 3 independent experiments (each performed in triplicate) and are represented as absorbance 595 nm optical density (O.D.). **B** Colony-forming ability (colony-forming units CFU) of Mewo and NIH-3T3 NAMPT over-expressing in comparison with control cells for 12 days. Cells were stained with crystal violet and representative images are shown. On the right, histograms show the cumulative quantification of the percentage (%) area with colonies at the end of the 12-days period (3 independent experiments performed in triplicates). Data in the Figure are presented as the mean ± SEM. Statistical significance was calculated using Mann–Whitney test. Significance was represented as: *p ≤ 0.05, **p ≤ 0.01, ***p ≤ 0.001, ****p ≤ 0.0001

To directly determine the pro-tumorigenic effect of NAMPT we over-expressed the enzyme in the melanoma Mewo cell line (*BRAF*-WT) and in NIH-3T3 (murine immortalized fibroblasts), very commonly exploited in this kind of experiments [31]. When testing the impact of NAMPT over-expression on their growth capacity, we observed that both Mewo and NIH-3T3 cells with NAMPT over-expression grew faster, as measured using the MTT assay (Fig. 7A). Loss of contact inhibition and high saturation density leading to uncontrolled proliferation are well-known features of transformed or malignant cells. In addition, by using colony-forming assays, we demonstrated that NAMPT over-expression leads to copious focus (colony-forming units, CFU) formation in Mewo and NIH-3T3 cells (Fig. 7B).

Together, these data suggest that NAMPT over-expression positively influences the capacity of melanoma cells to grow even independently from the mutations on the key oncogene BRAF. Additionally, our results on fibroblasts, paved the way to further studies to consolidate the notion to consider NAMPT a novel proto-oncogene in tumor.

## Discussion

The combination of BRAFi and MEKi has shown clinical activity in MM [15–17, 32], but also in other cancers carrying *BRAF* mutations, such as THCA (specifically, the anaplastic subtype) and NSCLC [33]. On the contrary, *BRAF*-mutated CRC demonstrated a relative resistance to BRAFi, suggesting that responses may be tumor/histology dependent [33, 34]. The rationale to combine BRAFi and MEKi was to delay the MAPK-driven acquired resistance, resulting in significant improvement in response rates, progression-free survival and overall survival, and decreasing the toxicities due to paradoxical activation of the MAPK pathway, observed with BRAF inhibitor monotherapy in MM [33, 35]. However, relapse and resistance continue to occur in many patients [36], prompting investigation into alternative or complementary therapeutic strategies, such as the combination with immunotherapy [37, 38]. Driven by paradoxical reactivation of MEK/ERK pathway downstream of BRAF [39], resistance to targeted therapy was extensively studied in *BRAF*-mutated MM leading to cellular reprogramming at multiple levels, including metabolism and epigenome organization, promoting a dynamic deregulation of differentiation/mesenchymal/stemness transcriptional programs [18, 19, 40–42].

Recently, we and others demonstrated that NAMPT is one of the main players in driving BRAFi resistance and melanoma plasticity, mediating metabolic adaptation, reshuffling of the epigenetic landscape and forcing an invasive/mesenchymal/stemness phenotype [8, 9, 11]. Moreover, we reported that BRAFi-resistant tumors were completely sensitive to NAMPTi in xenograft model [9].

*NAMPT* gene expression is finely tuned at the transcriptional levels by MYC, HIF-1α, STAT3-5, and NF-KB, connecting BRAF mutational signature to increased NAMPT levels [9, 11, 29]. However, a direct association between NAMPT and *BRAF* mutations at mRNA and protein levels was never highlighted. By exploiting engineered cell lines expressing a *BRAF* V600E construct, the TCGA database, and high-density tissue microarrays of a large collection of MM and THCA samples we demonstrated a positive correlation between mutations in the *BRAF* oncogene and NAMPT over-expression in tumors. These observations imply that the MAP kinase pathway regulates NAMPT expression, opening to further studies on the functional role of this enzyme not only in MM, but also in other tumors relying on this pathway for growth and survival.

To date the only evidence connecting mutations in oncogenic genes and sensitivity to NAMPT inhibition concerns tumors carrying isocitrate dehydrogenase (*IDH*) mutations, which are profoundly sensitive to NAD depletion through NAMPT inhibition [43]. Our data indicate that also tumors harboring *BRAF* mutations are uniquely sensitive to NAMPT inhibition and could therefore benefit from the introduction of NAMPTi in clinical settings. The identification of a subset of tumors (BRAF^mut^/NAMPT^high^) sensitive to NAMPT inhibition opens the way for a second life for NAMPTi, possibly to be used in combination with BRAFi/MEKi. We could envisage in the clinical practice to screen for *BRAF* mutations and for NAMPT expression by IHC, to identify the subset of patients with BRAF mutations and high NAMPT expression to be treated with a combination of NAMPTi in association with BRAFi/MEKi to postpone and/or overcome drug resistance.

The last piece of evidence of this paper supports a direct oncogenic role for NAMPT in tumors. Firstly, *NAMPT* gene was found amplified in several tumors with a direct correlation to its expression. In melanoma, we confirmed *NAMPT* amplification, as recently reported by Chowdhry et al. [29]. Furthermore, in agreement with these authors, we showed that MM is a type of tumor completely addicted to NAMPT, without significant amplification of *NAPRT*, as reported in other tumors [29]. Secondly, we found that the simple over-expression of NAMPT in BRAF wt MM cell line and in fibroblasts promotes an increased cell growth capacity. Overall, these findings, despite the need of further investigation, support the hypothesis that NAMPT amplification and over-expression may be a key event in tumorigenesis before or after the acquisition of driving mutations in other oncogenes and/or in tumor suppressor genes. For example, it is reasonable remind as in melanoma model, although mutated BRAF proteins possess elevated kinase activity and induce transformation in NIH-3T3 cells, oncogenic activation of *BRAF* V600E in melanocytes in vitro is insufficient for full malignant conversion due to activation of the protective mechanism of senescence [44]. This event could be bypassed during tumorigenesis in patients by concomitant mutations in *BRAF* oncogene and amplification/overexpression of NAMPT, as here shown.

Considered together, these data shed light on the molecular connection between *BRAF* mutations and expression of NAMPT, a key enzyme involved in NAD biosynthesis and metabolic reprogramming, revealing a novel function for it as proto-oncogene in tumors that merits further investigations.

## Conclusions

Our findings demonstrate that NAMPT is over-expressed in *BRAF*-mutated tumors and functionally linked to the activation of the BRAF oncogenic pathway. This molecular relationship makes *BRAF*-mutated tumors particularly sensitive to NAMPT inhibition and strongly encourages future clinical investigation of NAMPTi plus BRAFi/MEKi in MM and in tumors rely on MAPK pathway activation. Lastly, our results support a novel finding to consider NAMPT itself a proto-oncogene in cancer driving cellular transformation.

## Supplementary Information

**Additional file 1.** Supplemental methods. Details about TCGA datasets, Immunohistochemistry staining, cell culture and list of antibodies and primers used.** Figure S1.** Analysis of NAMPT expression in different tumors *BRAF* wild-type or *BRAF* mutated and analysis of *BRAF* mutations frequency in tumors.** Figure S2.** Comparison of sensitivity to NAMPT inhibitors between MM and THCA cells lines *BRAF*-mutated.** Figure S3.** Correlation between *NAMPT* copy number alterations (CNA) and NAMPT expression in tumors.

## Data Availability

All methods and materials used are described in the manuscript and data can be obtained from the corresponding authors upon reasonable request.

## Abbreviations

NAMPT: Nicotinamide phosphoribosyltransferase
MM: Metastatiuc melanoma
THCA: Thyroid carcinoma
TCGA: The Cancer Genome Atlas
NAD: Nicotinamide adenine dinucleotide
NBE: NAD-biosynthetic enzymes
TF: Transcription factors
CREB: CAMP response element-binding protein
STAT: Signal transducer and activator of transcription
HIF: Hypoxia inducible factor
NF-kB: Nuclear factor-kB
FFPE: Formalin-fixed paraffin embedded
