# Supplementary material for: Tumors carrying BRAF-mutations over-express NAMPT that is genetically amplified and possesses oncogenic properties
Source: J Transl Med. 2022 Mar 10;20:118. doi: 10.1186/s12967-022-03315-9 (PMC8908704; doi:10.1186/s12967-022-03315-9)
Supplement: Supplementary file 1 — Additional file 1. Supplemental methods. Details about TCGA datasets, Immunohistochemistry staining, cell culture and list of antibodies and primers used. Figure S1. Analysis of NAMPT expression in different tumors BRAF wild-type or BRAF mutated and analysis of BRAF mutations frequency in tumors. Figure S2. Comparison of sensitivity to NAMPT inhibitors between MM and THCA cells lines BRAF-mutated. Figure S3. Correlation between NAMPT copy number alterations (CNA) and NAMPT expression in tumors. [file 12967_2022_3315_MOESM1_ESM.docx]

**Title: Tumors carrying *BRAF*-mutations over-express NAMPT that is genetically amplified and possesses oncogenic properties.**

**Authors:** Valentina Audrito^1,§^, Enrico Moiso^2,3^, Filippo Ugolini^4^, Vincenzo Gianluca Messana^1^, Lorenzo Brandimarte^1^, Ilaria Manfredonia^1^, Simonetta Bianchi^4^, Francesco De Logu^4^, Romina Nassini^4^, Anna Szumera-Ciećkiewicz^5,6^, Daniela Taverna^7^, Daniela Massi^4^, and Silvia Deaglio^1,^*

**Affiliations:** ^1^Functional Genomic Unit, Department of Medical Sciences, University of Turin, Turin, Italy; ^2^Koch Institute for Integrative Cancer Research, Massachusetts Institute of Technology, Cambridge, MA, USA. ^3^Broad Institute of Harvard and MIT, Cambridge, MA, USA. ^4^Department of Health Sciences, University of Florence, Florence, Italy. ^5^Department of Pathology and Laboratory Medicine, Maria Sklodowska-Curie National Research Institute of Oncology, Warsaw, Poland. ^6^Diagnostic Hematology Department, Institute of Hematology and Transfusion Medicine, Warsaw, Poland. ^7^Molecular Biotechnology Center & Dept. Molecular Biotechnology and Health Sciences, University of Torino, Italy.

^§^Present address: Molecular Biotechnology Center & Dept. Molecular Biotechnology and Health Sciences, University of Torino, Italy

**Keywords:** NAMPT, oncogene, transformation, BRAF oncogenic signaling, MAPK, NAMPT inhibitors

***Corresponding author:** Silvia Deaglio, MD, PhD, Laboratory of Functional Genomics, Department of Medical Sciences, University of Turin, Via Nizza, 52, 10126 Torino, Italy. Phone number: +390116709535; +390116706497. Emails: silvia.deaglio@unito.it.

**Additional Methods**

*TCGA analysis*

Clinical and genomic data of TCGA cancer samples and patients (release 2016_02_28) were downloaded from the Broad TCGA GDAC site (https://gdac.broadinstitute.org/), by means of firehose get Version: 0.4.1. The data refers to a cohort of 11158 cancer patients of 33 different tumor types (Coding Genes Expression: RNAseqV2_RSEM_genes_normalized__data_Level_3, Mutation data: [Mutation_Packager_Oncotated_Calls](http://gdac.broadinstitute.org/runs/stddata__2016_01_28/data/ACC/20160128/gdac.broadinstitute.org_ACC.Mutation_Packager_Oncotated_Calls.Level_3.2016012800.0.0.tar.gz)), and Copy Number Alteration (CNA) data: derived from Level_3_segmented_scna_minus_germline_cnv.

*Immunohistochemistry staining*

*Tissues samples*

The samples included in the study were identified in the databases from 2010 to 2019. The use of Formalin-fixed and paraffin-embedded (FFPE) sections of human samples was approved by the Local Ethics Committee (16275_bio) according to the Helsinki Declaration. FFPE tissue sections, 3 μm in thickness, were stained with hematoxylin-eosin and reviewed to confirm the histopathological diagnosis and pathology tissue quality control.

*Immunohistochemistry*

FFPE whole slides sections of THCA were stained with BRAF V600E antibody (mouse monoclonal, clone VE1 ready to use; Ventana Medical Systems, Tucson, AZ) and then representative sections (n=84) were selected for NAMPT staining. After pretreatment with CC1, TMAs and tissue sections were incubated with primary antibodies on a Ventana Benchmark XT immunostainer. The signal was developed with the UltraMap DAB anti-Mouse Detection Kit (Ventana Medical Systems, Tucson, AZ). Sections were counterstained with hematoxylin.

*NAMPT evaluation (H-score)*

A semi-quantitative H-score analysis of NAMPT staining in *BRAF*-mutated and *BRAF*-WT cases of MM and THCA was performed. Briefly, the percentage of NAMPT positive cells (0% to 100%) was multiplied by the dominant intensity pattern of staining (1, weak; 2, moderate; 3, strong), with an overall score ranging from 0 to 300. In this system, <1% positive cells were considered a negative result.

*Cell Culture*

Cell lines were cultured in RPMI-1640 (Sigma, Milan, IT) or Dulbecco's Modified Eagle Medium (DMEM, from Thermo Fischer Scientific, Rodano MI, IT) supplemented with 10% of fetal calf serum (FCS), and 10 IU/ml of penicillin / streptomycin (all from Sigma). Cell lines were tested to confirm lack of mycoplasma contamination.

*TaqMan Gene Expression Assays*

The TaqMan Gene Expression Assays (Thermo Fisher Scientific) used were: Hs00237184_m1 (*NAMPT*), Hs00944470_m1 (*NMRK1*), Hs00376971_g1 (*NAPRT*), Hs00204757_m1 (*QPRT*) and Hs99999903_m1 (*ACTB*), used as housekeeping gene.

*Antibodies used for Western blot analysis*

Antibodies used were: anti-*BRAF* V600E (RM8, ab200535, Abcam, Cambridge, UK), anti-NAMPT (A300-779A, Bethyl Laboratories, Montgomery, TX), anti‐phospho-ERK1/2 (pT202/pY204, #612359), anti-pan-ERK1/2 (#610124) both from BD Biosciences (San Jose, CA), anti-β-Actin (sc-47778) from Santa Cruz Biotechnology (Dallas, TX). Secondary reagents were: goat anti‐mouse IgG‐HRP-conjugated (Perkin Elmer, Monza, IT) and goat anti‐rabbit HRP‐conjugated (Santa Cruz Biotechnology).

**Additional Figures and legends**

**
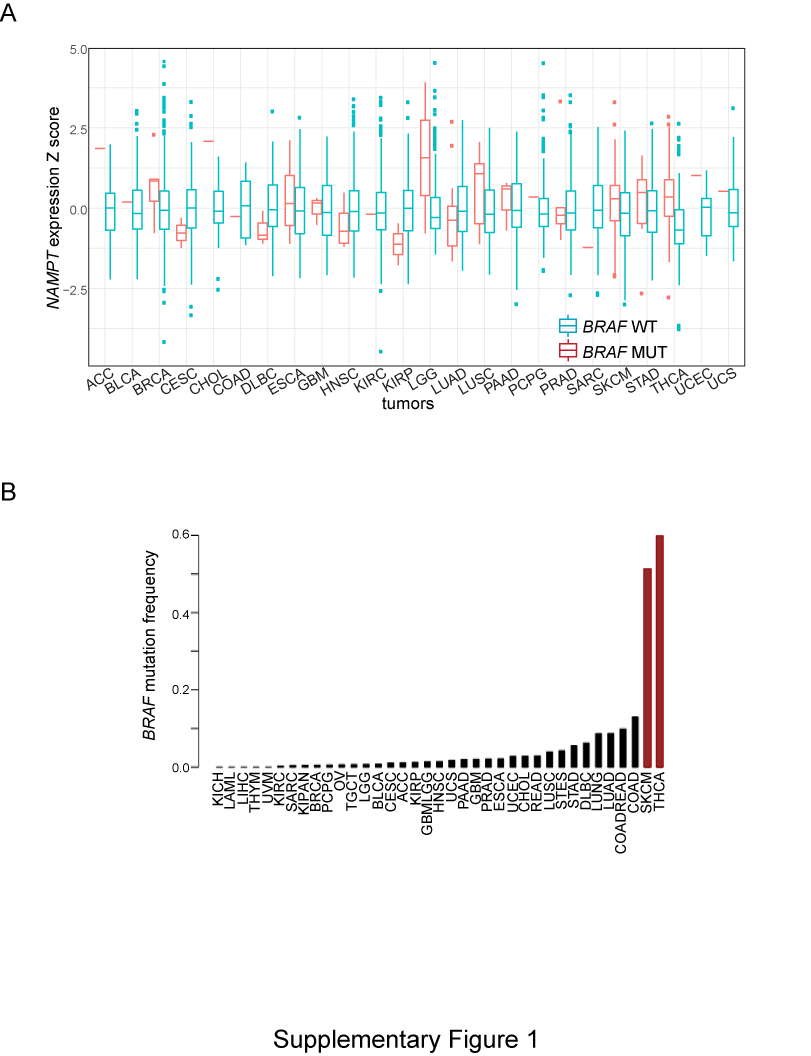

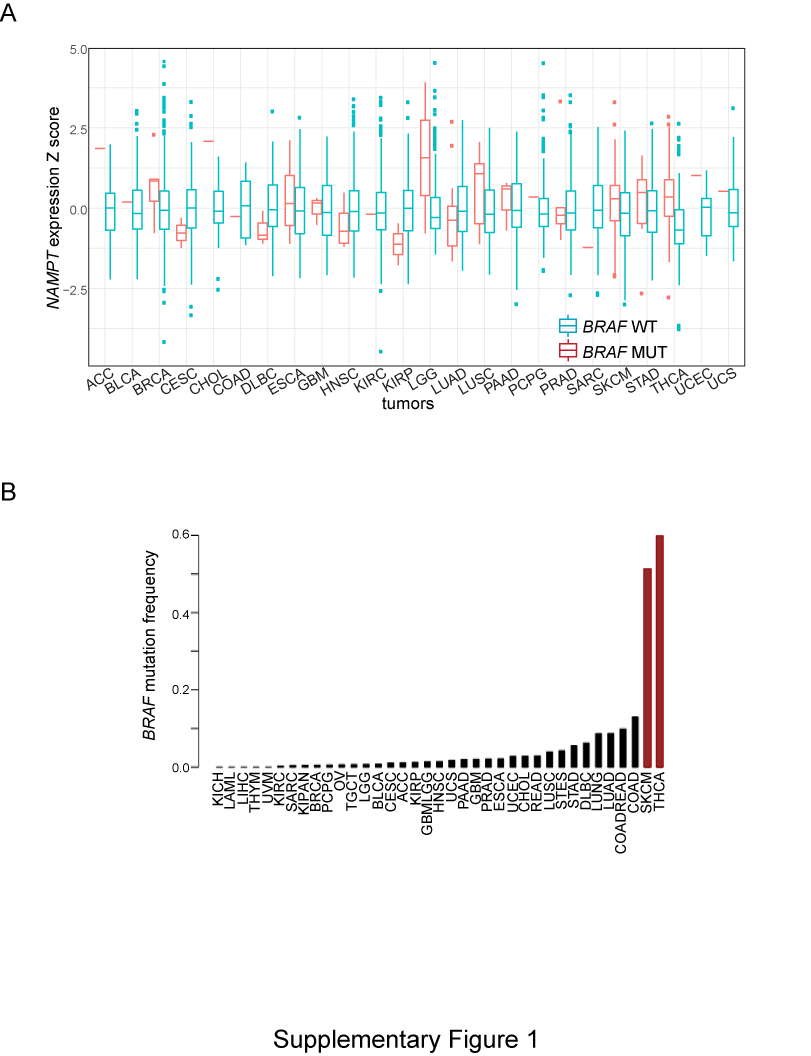
**

C

B

A


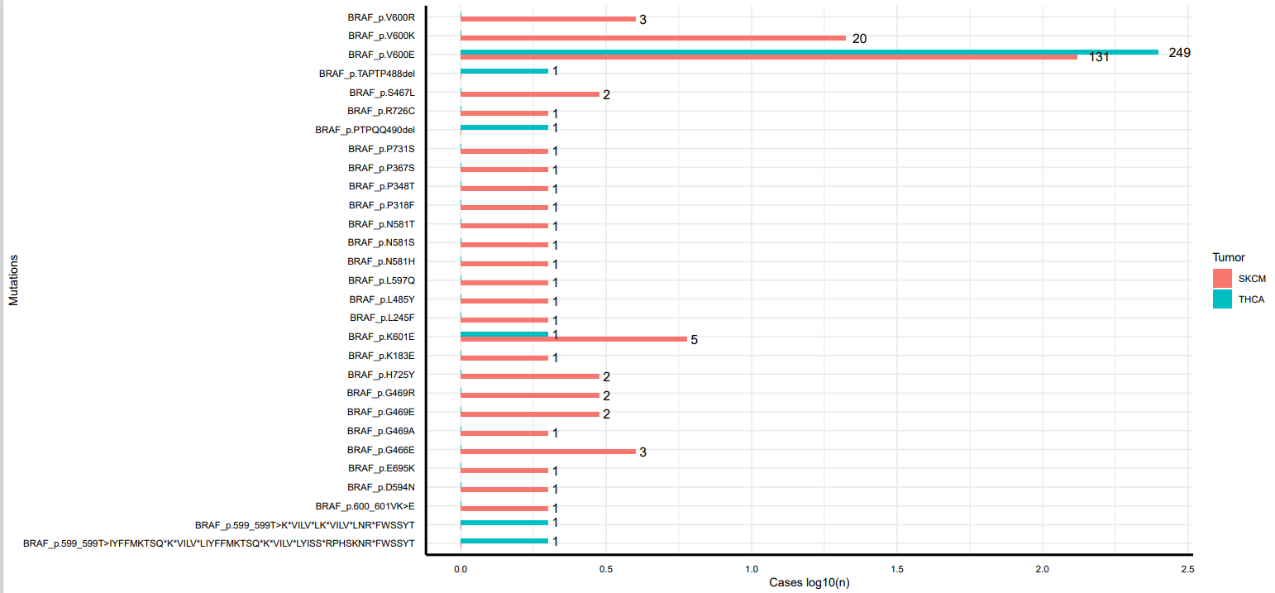


**Fig. S1. *NAMPT* expression is associated with *BRAF* mutations in TCGA cohort. (A)** Boxplots representing *NAMPT* expression per tumor type in the TCGA cohort, represented as Z score. Complete list of TCGA abbreviations is available at https://gdc.cancer.gov/resources-tcga-users/tcga-code-tables/tcga-study-abbreviations. **(B)** Bar-plot representing *BRAF* mutation frequency in the whole TCGA cohort. Melanoma (SKCM) and thyroid carcinoma (THCA) samples harbor the highest number of mutations (red bars). (**C**) Histogram plot showing the distribution of the different BRAF mutations in the patient cohort analyzed.

**
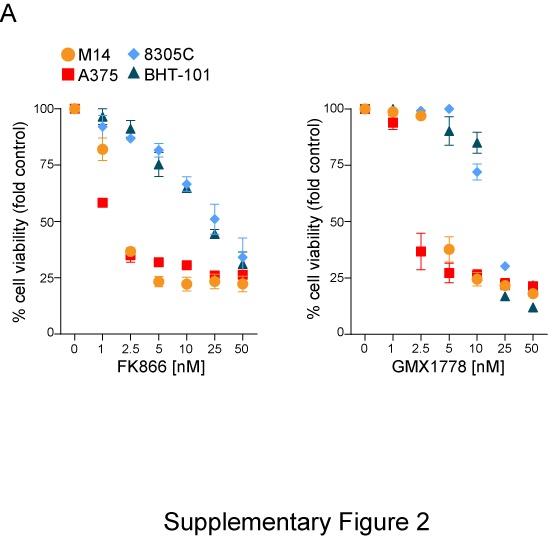
**

**Fig. S2. Response to NAMPT inhibition in MM and THCA cell lines according to *BRAF* mutations.** **(A)** Short-term proliferation assay of MM *BRAF*-mutated cell lines (shades of red) and THCA *BRAF*-mutated cell lines (shades of blue). Cells were treated for 72 hours with increasing concentrations of two NAMPTi (FK866 and GMX1778) and then cell viability was measured using MTT assay. Data represent the average and SEM of at least three biological replicates. Data were represented as % of cell viability over untreated condition. First data points of the curves represent the control.


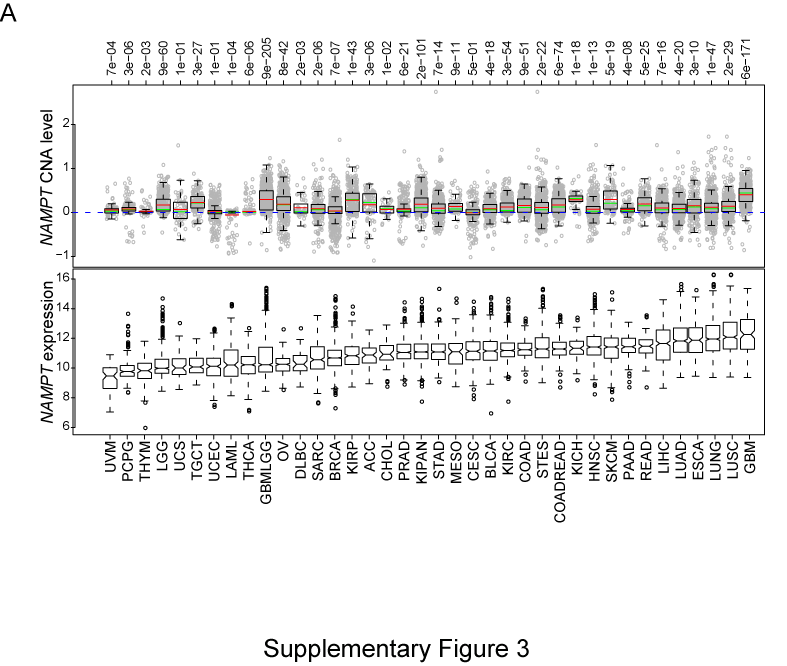


**Fig. S3. *NAMPT* copy number alterations (CNA) correlates with *NAMPT* expression in tumors. (A)** Boxplots representing the detail of *NAMPT* CNA (upper panel) score and *NAMPT* expression (lower panel) per tumor type in the TCGA cohort. Complete list of TCGA abbreviations is available at https://gdc.cancer.gov/resources-tcga-users/tcga-code-tables/tcga-study-abbreviations. Tumor types are sorted by *NAMPT* expression levels. p-values on the upper panel represent the significance of the difference between the mean CNA score from zero in every tumor type.
